# Macrophage-Secreted Lipocalin-2 Promotes Regeneration of Injured Primary Murine Renal Tubular Epithelial Cells

**DOI:** 10.3390/ijms21062038

**Published:** 2020-03-16

**Authors:** Anja Urbschat, Anne-Kathrin Thiemens, Christina Mertens, Claudia Rehwald, Julia K. Meier, Patrick C. Baer, Michaela Jung

**Affiliations:** 1 Department of Biomedicine, Aarhus University, 8000 Aarhus, Denmark; anja.urbschat@biomed.au.dk; 2 Division of Nephrology, Department of Internal Medicine III, Goethe-University Frankfurt, 60323 Frankfurt am Main, Germany; Anne-Kathrin.Thiemens@web.de (A.-K.T.); p.baer@em.uni-frankfurt.de (P.C.B.); 3 Institute of Biochemistry I, Goethe-University Frankfurt, Faculty of Medicine, 60323 Frankfurt am Main, Germany; Christina.Mertens@med.uni-heidelberg.de (C.M.); rehwald@biochem.uni-frankfurt.de (C.R.);

**Keywords:** renal tubular epithelial cells, macrophages, lipocalin-2, iron

## Abstract

Lipocalin-2 (Lcn-2) is rapidly upregulated in macrophages after renal tubular injury and acts as renoprotective and pro-regenerative agent. Lcn-2 possesses the ability to bind and transport iron with high affinity. Therefore, the present study focuses on the decisive role of the Lcn-2 iron-load for its pro-regenerative function. Primary mouse tubular epithelial cells were isolated from kidney tissue of wildtype mice and incubated with 5 µM Cisplatin for 24 h to induce injury. Bone marrow-derived macrophages of wildtype and Lcn-2^−/−^ mice were isolated and polarized with IL-10 towards an anti-inflammatory, iron-release phenotype. Their supernatants as well as recombinant iron-loaded holo-Lcn-2 was used for stimulation of Cisplatin-injured tubular epithelial cells. Incubation of tubular epithelial cells with wildtype supernatants resulted in less damage and induced cellular proliferation, whereas in absence of Lcn-2 no protective effect was observed. Epithelial integrity as well as cellular proliferation showed a clear protection upon rescue experiments applying holo-Lcn-2. Notably, we detected a positive correlation between total iron amounts in tubular epithelial cells and cellular proliferation, which, in turn, reinforced the assumed link between availability of Lcn-2-bound iron and recovery. We hypothesize that macrophage-released Lcn-2-bound iron is provided to tubular epithelial cells during toxic cell damage, whereby injury is limited and recovery is favored.

## 1. Introduction

Despite enormous advances in treatment of patients suffering from acute kidney injury, we still encounter a high morbidity and mortality rate. Hence, effective approaches for prevention and treatment are still lacking [1,2], with only limited and unsatisfactory therapeutic options. Fortunately, the kidney has the intrinsic capacity to recover from ischemic or toxic insults that cause renal cell death [3]. Therefore, timely rescue of affected renal tubules may arrest progression of injury and pave the way for recovery. The response to acute kidney injury involves a complex network of interconnected and orchestrated mechanisms. Herein, macrophages (MΦ) constitutes one of the major infiltrating cell populations in acute renal injury [4,5]. Moreover, MΦ-infiltration was recognized as a crucial feature of both the initial severity of injury and progression of renal failure, but also following regeneration and repair phase [6]. In this regard, MΦ shows a remarkable repertoire of functional and phenotypic activation states [7,8]. In particular, during the acute phase of tissue injury, MΦ shows a pro-inflammatory phenotype, whereas they adopt an anti-inflammatory phenotype during later phases of tissue recovery [9,10,11,12]. This enables them to determine the fine balance of injury versus regeneration of damaged renal tissue. Accordingly, we found that MΦ genetically modified ex vivo in order to express a predetermined anti-inflammatory phenotype showed a clearly protective role upon re-infusion into ischemic kidneys [10]. Therefore, it might be speculated that the control of the local MΦ phenotype plays a decisive role for the inflammatory outcome and, thus, disease progression.

We recently described lipocalin-2 (Lcn-2) as a potent determent of MΦ polarization in the context of kidney injury [9]. Lcn-2 itself is a 25 kDa protein of the lipocalin superfamily that is rapidly upregulated after renal tubular injury [13,14]. It represents both a biomarker of renal ischemic injury [15] including Cisplatin injury [16] and a renoprotective agent when exogenously administered [17,18,19]. Lcn-2 is expressed in MΦ upon contact to apoptotic cells [11,20,21]. Mechanistically, the induction of Lcn-2 was stimulated by apoptotic cell-secreted sphingosine-1-phophate (S1P) and the downstream activation of the STAT3 signaling pathway [20].

Though, using a neutralizing antibody approach, we previously were able to show that Lcn-2 plays a pivotal role during injury, but also in the reparative phases of ischemia reperfusion injury. Intriguingly, Lcn-2-mediated cell regeneration was dependent on the inflammatory micromilieu of the tissue [22]. Moreover, the infusion of Lcn-2-overexpressing macrophages significantly increased renal epithelial cell proliferation. This effect was blocked by Lcn-2 neutralizing antibodies or the infusion of MΦ with a knockdown of Lcn-2 [9]. However, it was previously described that exclusively iron-loaded Lcn-2 triggers cell survival upon internalization [23]. Thus, the functional outcome of Lcn-2 seems to largely depend on its iron-load. It was already speculated that the beneficial effect of Lcn-2 against ischemia reperfusion injury to the kidney may be a result of its ability to bind and transport iron to viable cells, thereby limiting cell death, promoting proliferation, and enhancing recovery [17,18,24,25]. Although its ability to transport and donate iron to cells seems to determine the pro-survival and/or anti-apoptotic function of Lcn-2, the decisive role of the iron-load of Lcn-2 has not been investigated so far in a Cisplatin-dependent nephrotoxicity model.

Given the pivotal role of Lcn-2 as well as MΦ for iron homeostasis and the fact that the presence of both was associated with increased renal regeneration upon injury, the current study aimed at investigating their interconnection. Considering that we previously observed that the administration of Interleukin (IL)-10-overexpressing MΦ induces Lcn-2 and its receptors in the kidney [10] and as Cisplatin is known to affect mainly the proximal tubular cells of the kidney [26,27,28], we performed a cell culture study with isolated primary murine renal tubular epithelial cells injured by various doses of Cisplatin. In order to better understand renal damage and repair mechanisms as well as for testing potential therapeutic options, post-injury recovery was favored through exposure to conditioned medium from either murine wildtype (wt) C57BL/6 or Lcn-2^−/−^ C57BL/6 bone marrow-derived MΦ (BMDM).

## 2. Results

### 2.1. Dose-Dependent Injury of Primary Mouse Tubular Epithelial Cells (mTECs) upon Incubation with Cisplatin

**Figure 1:**
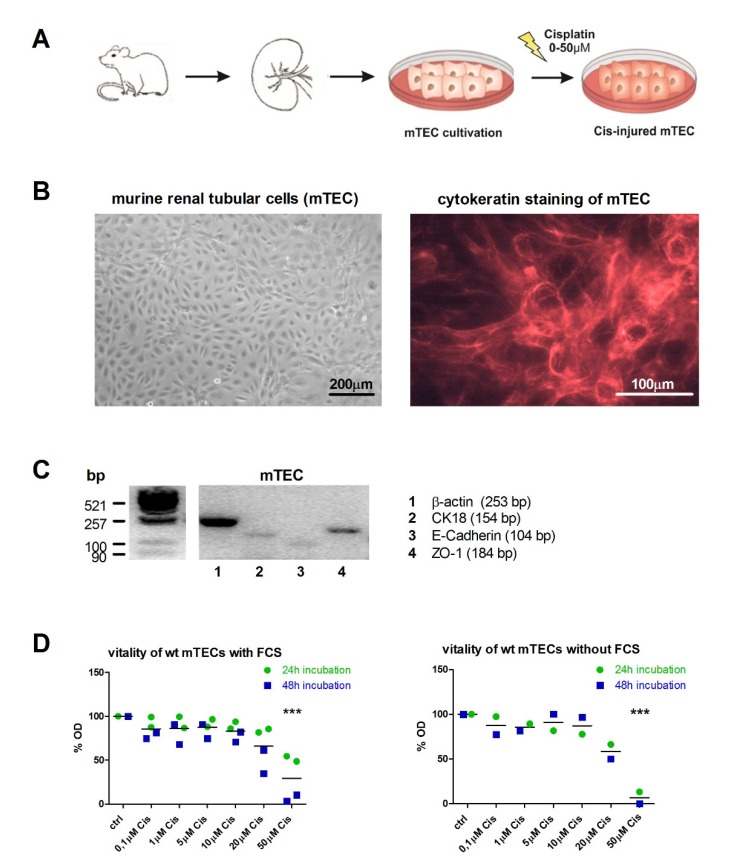
Primary murine tubular epithelial cells respond to Cisplatin-induced injury. (**A**) Schematic overview of experimental setup. (**B**) Cultured murine renal tubular epithelial cells (mTEC) in vitro displaying an epithelial morphology in culture (left) and stained positive for the epithelial cell marker cytokeratin 18 (right). (**C**) qPCR-analysis of epithelial cell markers cytokeratin 18 (CK18), E-Cadherin, and ZO-1 confirmed the epithelial origin of isolated primary mTEC. (**D**) Measurement of cell vitality via XTT assay using increasing Cisplatin concentrations: 0.1 µM, 1 µM, 5 µM, 10 µM, 20 µM, 50 µM, and untreated control (ctrl) for 24 h and 48 h, respectively. The untreated ctrl has been set on 100% of optical density (OD), and treated cells are given in percentage in relation to ctrl. *** *p* < 0.001 (*n* = 3, one-way ANOVA followed by Tukey’s Multiple Comparison Test).

Isolated primary mTECs of wt C57BL/6 mice displaying the morphology of renal tubular epithelial cells (Figure 1B, left) and were first stained with cytokeratin to prove their epithelial origin (Figure 1B, right). qPCR analysis of epithelial cell markers cytokeratin 18 (CK18), E-Cadherin, and zona occludens (ZO)-1 confirmed the tubular epithelial origin of isolated mTECs (Figure 1C). In order to evaluate the appropriate Cisplatin concentration, which injured the cells, but still allowed cellular recovery, we measured cell vitality via XTT assay using increasing Cisplatin concentrations: 0.1 µM, 1 µM, 5 µM, 10 µM, 20 µM, and 50 µM compared to untreated controls (ctrl) for 24 h and 48 h, respectively (Figure 1D). This has been performed twice with in total 9 technical replicates with 5% FCS and confirmed once with 6 technical replicates without FCS. As expected, vitality significantly decreased with increasing Cisplatin concentrations. mTECs cultured with FCS (Figure 1D, left) represented overall higher levels of mTEC vitality than mTECs cultured without FCS (Figure 1D, right). A significant difference between 24 h or 48 h of Cisplatin treatment was not observed for none of the experimental groups. Based on these results, we used 5 µM Cisplatin without FCS for all following experiments, as this turned out to represent the most adequate dosage, displaying measurable injury parameters, but moderate cell injury.

### 2.2. Establishment of Conditioned Media and Macrophage Polarization

**Figure 2:**
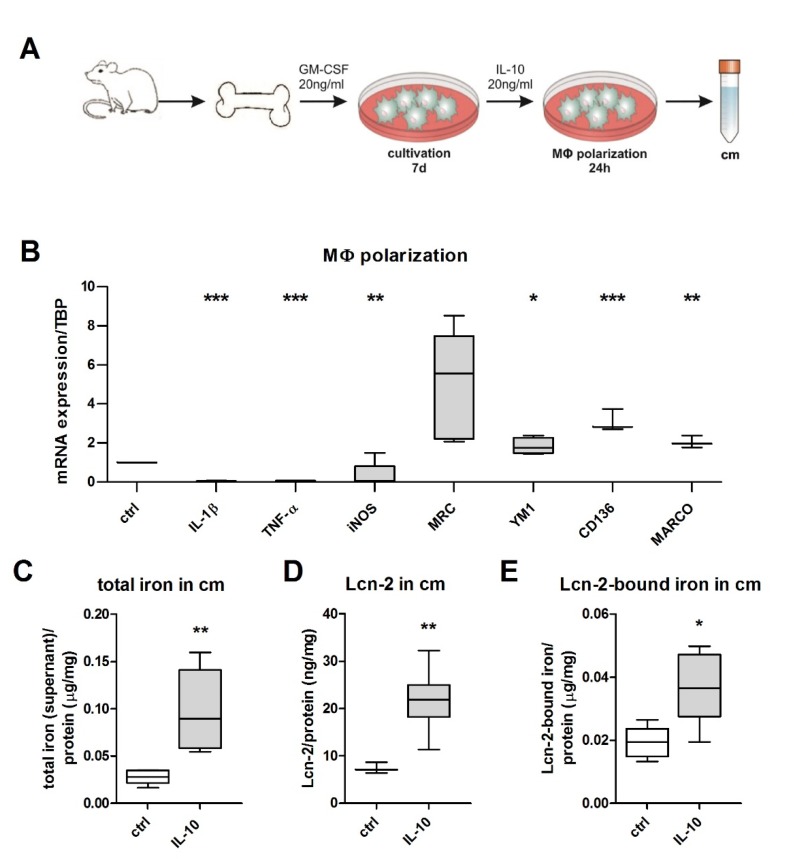
Primary bone marrow-derived macrophages (BMDM) release Lcn-2-bound iron upon IL-10 treatment. (**A**) Schematic representation for the generation of conditioned media from BMDMs. (**B**) qRT-PCR analysis of IL-10-stimulated macrophages compared to untreated control relative to the housekeeping gene TBP (*n* = 5; *t*-test). (**C**) Total iron amount in the supernatant of both wt and Lcn-2^−/−^ BMDM measured by atomic absorption spectrometry (AAS). (**D**) Measurement of Lcn-2 protein in macrophage conditioned media via ELISA. (E) Immunoprecipitation of Lcn-2 with subsequent iron measurements via AAS to determine the amount of Lcn-2-bound iron in macrophage conditioned media. * *p* < 0.05, ** *p* < 0.01, *** *p* < 0.001 (*n* = 5; *t*-test).

We next isolated primary murine MΦ from wt and Lcn-2^−/−^ mice and stimulated them with IL-10 (20 ng/mL) for 24 h to induce an anti-inflammatory activated as well as iron-releasing phenotype. The resulting conditioned medium (cm) was collected for further analysis and subsequent stimulation of mTEC. A schematic representation of the experimental set-up is given in Figure 2A. qRT-PCR analyses of polarized MΦ show low expression of pro-inflammatory markers IL-1β (Interleukin 1β) (*p* < 0.01; *n* = 5), TNF-α (tumor necrosis factor) (*p* < 0.01; *n* = 5), and iNOS (inducible nitric oxide synthase) (*p* < 0.05; *n* = 5), whereas the anti-inflammatory markers MRC (macrophage mannose receptor) (ns), Ym1 (chitinase 3-like 3), CD163, and MARCO (macrophage receptor with collagenous structure) were elevated compared to untreated control MΦ (Figure 2B). In order to verify the iron-releasing MΦ phenotype, we determined the iron amount in the supernatant via atomic absorption spectroscopy (AAS) relative to the total protein content, showing a significant increase in relative iron levels in IL-10-treated MΦ supernatants (Figure 2C, *p* < 0.01; *n* = 5). As previously observed for human MΦ [29], we also observed an increased release of Lcn-2 to the supernatant of IL-10-stimulated murine MΦ (Figure 2D). In order to determine the amount of iron bound to Lcn-2 in MΦ supernatants, we performed an immunoprecipitation for Lcn-2 with subsequent measurement of the Lcn-2-bound iron applying AAS of immunoprecipitated samples. Interestingly, Lcn-2-bound iron was significantly elevated in IL-10-treated MΦ supernatants (Figure 2E, *p* < 0.05; *n* = 5). 

### 2.3. Conditioned Medium From wt MΦ or the Supply of Holo-Lcn-2 Tends to Promote Epithelial Viability upon Cisplatin Treatment

**Figure 3:**
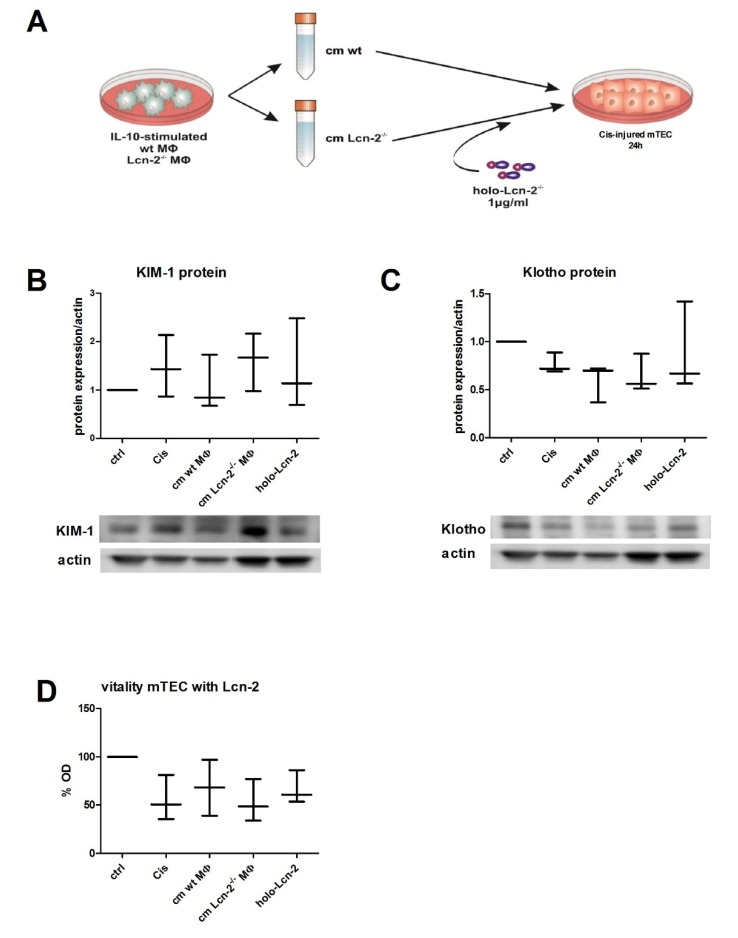
Cellular vitality depends on the presence of Lcn-2-bound iron in macrophage supernatants. (**A**) Visualization of the experimental set up. mTECs were either cultivated with standard medium without FCS (ctrl) or injured by 24 h incubation with 5 µM Cisplatin (Cis). Hereafter, Cisplatin-injured mTECs were cultured 24 h further in conditioned medium from IL-10-stimulated MΦ from wt mice (wt cm) or Lcn-2^−/−^ mice (Lcn-2^−/−^ cm). The latter group was further cultivated with supplementation of iron-containing holo-Lcn-2 (designated as Lcn-2^−/−^ cm + holo-Lcn-2). (**B**) Kidney injury molecule 1 (KIM-1) (44kDA) and (**C**) Klotho (130kDA) protein expression relative to β-actin (ns; *n* = 3). (**D**) Measurement of cellular vitality after cm-treatments via XTT (ns; *n* = 3).

We next aimed at determining the influence of MΦ-released Lcn-2, especially in its iron-loaded form, on mTEC recovery after Cisplatin-induced injury. In Figure 3A, the experimental set-up is visualized. mTECs were either cultured with standard medium without FCS (ctrl) or injured with 5 µM Cisplatin both for 24 h and left without further treatment (Cis) or treated with cm for an additional 24 h, deriving either from IL-10-treated wt (wt cm) or Lcn-2^−/−^ MΦ (cm Lcn-2^−/−^). We then measured the expression of kidney injury molecule 1 (KIM-1), a well-accepted acute injury marker of the proximal tubule system, also in Cisplatin-induced renal injury both in vitro and in vivo [30]. KIM-1 protein expression increased upon Cisplatin treatment and was lowered to control levels upon incubation of mTEC with the supernatant of wt MΦ, showing a trend towards the recovery of mTECs (ns). In contrast, the addition of Lcn-2^−/−^ cm did not decrease KIM-1 expression of mTECs. However, the addition of recombinant, iron-loaded Lcn-2 (holo-Lcn-2) to Lcn-2 lacking cm appeared to reduce KIM-1 expression (Figure 3B, *n* = 3, ns). We next analyzed the expression of Klotho, as it is highly expressed in the healthy kidney and known to significantly decline upon kidney injury but rises gradually to control level during recovery [31,32,33]. Accordingly, we observed a conversely trajectory compared to KIM-1, with a decrease of Klotho protein expression upon cisplatin treatment (ns). However, we observed no changes of Klotho expression upon cm treatment, neither from wt nor from Lcn-2^−/−^ MΦ (Figure 3C, *n* = 3, ns). We obtained similar results as for Klotho expression for viability test via XTT, again lacking statistical significance (ns; *n* = 3) (Figure 3D).

**Figure 4:**
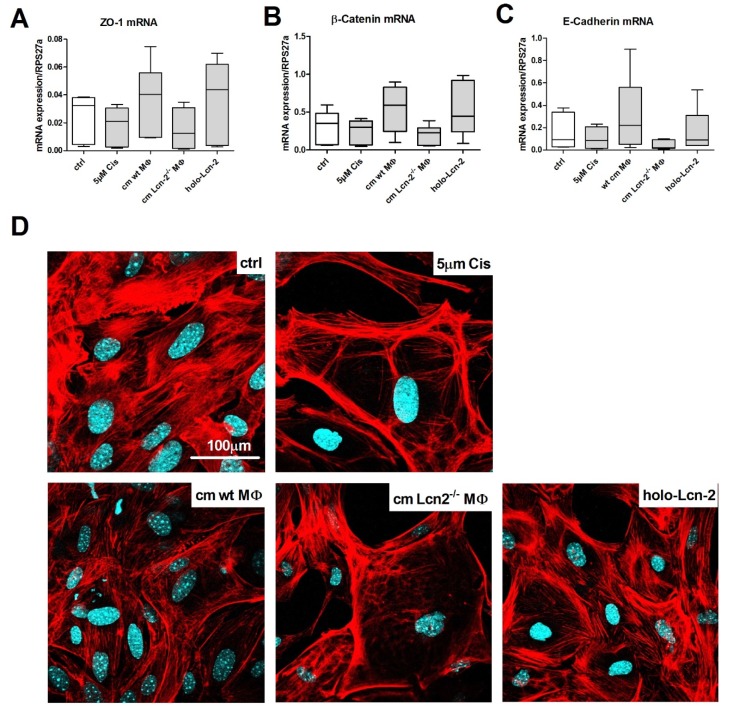
The epithelial phenotype is rescued by Lcn-2. mRNA expression relative to the housekeeping gene RPS27a of the epithelial phenotype markers (**A**) Zonula occludens-1 (ZO-1), (**B**) β-catenin, and (**C**) E-Cadherin (ns; *n* = 6). (**D**) Phalloidin staining visualizing the cytoskeleton and the F-actin stress fibers in red and nuclei in blue.

Furthermore, we analyzed the expression of epithelial cell markers ZO-1 (Figure 4A), β-catenin (Figure 4B), and E-Cadherin (Figure 4C) via qRT-PCR. Upon Cisplatin treatment, all three markers decreased, which could be rescued by the addition of wt cm, whereas Lcn-2^−/−^ cm did not induce recovery of mTECs. The addition of iron-loaded Lcn-2 induced the expression of epithelial markers, thereby rescuing the deleterious effect of Lcn-2^−/−^ cm on mTECs recovery profile (*n* = 6). This is further visualized by cytoskeletal integrity of mTECs applying phalloidin staining. Cisplatin-treated mTECs showed marked rarefaction of the cytoskeleton and junctional ring formation of F-actin fibers close to the surface. Upon incubation of Cisplatin-injured mTECs with wt cm, we observed a significant recovery of the cytoskeletal distribution across the cell, which was not observed upon stimulation with Lcn-2^−/−^ cm. Again, the addition of iron-loaded Lcn-2 significantly improved cytoskeletal integrity of damaged mTECs (Figure 4D).

### 2.4. Lcn-2-Mediated Iron Uptake Promote Proliferation of Cisplatin-Injured mTECs

**Figure 5:**
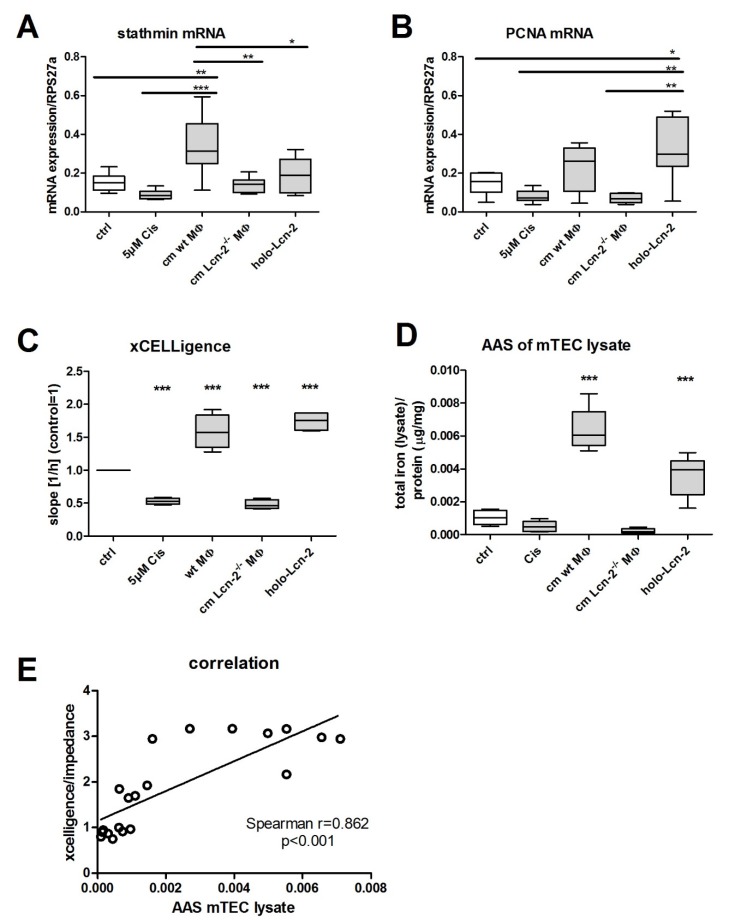
mTEC take up Lcn-2-bound iron that correlates with cellular proliferation. mRNA expression of (**A**) stathmin and (**B**) PCNA expression relative to the housekeeping gene RPS27a (*n* = 6). (**C**) Measurement of real-time proliferation via xCELLigence (*p* < 0.001; *n* = 5; one-way ANOVA followed by Tukey’s Multiple Comparison Test). (**D**) Measurement of total iron content in lysates of differently treated mTEC via AAS (*n* = 6). * *p* < 0.05, ** *p* < 0.01, *** *p* < 0.001. (**E**) Correlation between total iron amount in mTEC lysates and proliferation (all values included; Spearman r = 0.862, *p* < 0.001).

To investigate regenerative and proliferative parameters, we performed gene expression analyses on stathmin and proliferating-cell-nuclear-antigen (PCNA) gene expression, which are well-described markers in recovery from acute kidney injury [34,35]. Both stathmin (Figure 5A) and PCNA (Figure 5B) displayed decreased mRNA expression upon Cisplatin treatment and significantly increased upon treatment with wt cm treatment. In contrast, Lcn-2^−/−^ cm did not induce stathmin and PCNA expression in Cisplatin-injured mTECs, which could be rescued by the addition of recombinant holo-Lcn-2 protein. These observations were confirmed by proliferation measurements in real time up to 72 h (Figure 5C). Finally, we checked if mTEC take up MΦ-released iron and performed AAS-measurements of mTEC cellular lysates after cm-stimulation (Figure 5D, *n* = 6; *p* < 0.001). We observed a significant increase in intracellular iron amount upon treatment with wt cm, whereas the treatment with Lcn-2^−/−^ cm remained without effect. The addition of holo-Lcn-2 showed significantly enhanced intracellular iron levels in mTECs. The relevance of our findings could be reinforced by analyzing the correlation of intracellular iron amount and cellular proliferation (Figure 5E). Notably, the total iron content correlated with mTEC proliferation measured via xCELLigence (all values included; Spearman *r* = 0.862, *p* < 0.001).

As proof of concept that the delivery of iron supports mTEC recovery, we first measured the expression of KIM-1 as an injury marker (Figure S1A). Results show that the addition of the iron chelator 2′2 DPD (dipyridyl; 100 µM, 24 h) to wt cm treated mTEC increased the mRNA expression of KIM-1 (ns), indicating increased tubular damage. On the contrary, by measuring the epithelial markers E-Cadherin, β-catenin, and ZO-1, we observed a significant decrease (except for E-Cadherin) upon 2′2 DPD addition (Figure S1B). Moreover, proliferation markers PCNA and stathmin corroborated these observations (Figure S1C), showing significantly reduced expression.

**Figure 6:**
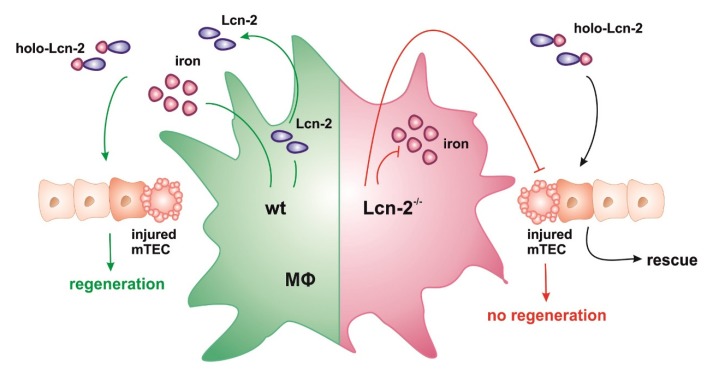
Results summary. Schematic overview of the hypothesis. After the stimulation of wt macrophages (MΦ) with IL-10, they adopt an iron-releasing phenotype, whereas Lcn-2^−/−^ macrophages do not release iron to the extracellular space. Wt MΦ also produce Lcn-2 in high numbers that chelates iron and donates these complexes to injured tubular epithelial cells (TEC), where recovery and regeneration are promoted. The addition of iron-containing holo-Lcn-2 rescues the negative effect of Lcn-2^−/−^ MΦ supernatants.

We therefore postulate that MΦ-released Lcn-2 binds and transports iron to damaged mTEC. In turn, the uptake of iron-Lcn-2 complexes facilitates tissue regeneration and promotes cellular proliferation. This hypothesis is depicted in Figure 6.

## 3. Discussion

The present study unravels a previously underappreciated function of MΦ-derived Lcn-2 in renal epithelial cell regeneration. Evidently, iron-loaded Lcn-2 promotes an epithelial phenotype integrity as well as cellular proliferation after Cisplatin-induced injury in vitro. A number of studies previously acknowledged the protective role of Lcn-2 in kidney injury, both in acute and chronic pathologies. Our current study adds to the emerging role of iron in determining the pro-regenerative function of Lcn-2 after renal injury, including ischemia/reperfusion injury (IRI) as well as nephrotoxic Cisplatin-induced damage.

Following kidney injury, renal tubular epithelial cells regenerate depending on the degree of damage or microenvironmental conditions [3]. Previous studies from our and other groups delineated a pivotal role for the MΦ phenotype influencing the inflammatory environment and determining kidney repair [10,11,36]. Besides, data from our group suggest that renal cell regeneration after mouse kidney IRI depends on endogenously generated MΦ-derived Lcn-2, whose expression is mainly affected by inflammatory cytokines [6,11]. Moreover, in a Cisplatin-induced rat renal injury model, Lcn-2 was found to be expressed in epithelial cells of the affected proximal renal tubules, whereby regeneration was promoted [37]. It is well established that the expression of Lcn-2 in renal tubular epithelial cells correlates to the degree of damage induced by either Cisplatin or IRI, whereby Lcn-2 serves as a biomarker and an acute phase protein. With regard to our previous observations as well as the results of the present study, it is therefore very important to distinguish the source of Lcn-2 as well as its iron-load in the kidney to determine its biological activity.

The infusion of exogenous Lcn-2 not only enhanced cellular proliferation, but also induced the expression of early progenitor markers in the kidney, thus suggesting that Lcn-2 might act as a growth and differentiation marker [19]. These observations are in line with results from the present study, showing that Lcn-2 enhanced epithelial integrity and polarity by inducing the expression of ZO-1, β-catenin, and E-Cadherin after Cisplatin-induced injury in primary mTEC. In line, it was previously appreciated that kidney injury results in loss of epithelial cell polarity, disruption of the actin cytoskeleton, and disassembly of junctional complexes [38,39]. In addition, one of our previous studies as well as Krudering et al. demonstrated that Cisplatin affects directly the cytoskeleton structure and causes apoptosis and cell detachment, resulting in a loss of F-actin fibers within a few hours of exposure [40,41]. We were able to confirm these morphological alterations in the present in vitro experiments: Cisplatin exposure clearly induced the rarefication and congestion of F-actin stress fibers. Yet, application of holo-Lcn2 led to recovery of F-actin fiber distribution across the cells and, thus, cellular integrity. In order to sustain these findings, we analyzed PCNA and stathmin as accepted regenerative markers in kidney injury [22,42,43]. Furthermore, the expression of both stathmin and PCNA mRNA is known to decrease following Cisplatin application in vivo [34], which is in line with our results in vitro. Previous own investigations showed that the proliferative markers Ki-67 and PCNA markedly increased in Lcn-2-overexpressing MΦ-treated rats after IRI [9]. However, blocking Lcn-2 production reduced the potency of a MΦ-based cell therapy approach, thereby substantiating the pro-proliferative and anti-inflammatory role of Lcn-2. In this regard, Kashiwagi et al. defined in their in vivo Cisplatin-induced rat kidney injury study that immunohistochemical expressions of Lcn-2 was mainly observed in regenerating renal tubules in the affected cortico-medullary junction and that the number of PCNA-positive cells significantly correlated with Lcn-2 scoring [37]. Taking these observations into account, the interplay between Lcn-2 and pro-regenerative MΦ holds potential in renal epithelial cell regeneration and hence, may define injury outcome.

Along these lines of evidence, it was previously recognized that anti-inflammatory polarized MΦ plays a crucial role during tissue repair, which is mainly achieved through phagocytosis of apoptotic cells and the subsequent production of anti-inflammatory mediators. Previous studies from our group indicate that sphingosine-1-phosphate (S1P) is released during apoptotic cell death and participates in coordinating anti-inflammatory responses in macrophages through the induction of Lcn-2 [11]. Bone-marrow-derived MΦ, engineered ex vivo to overexpress the anti-inflammatory cytokine IL-10, accumulated in IRI-damaged kidney tissue and promoted tissue regeneration. Interestingly, these infused cells showed enhanced iron levels as well as Lcn-2, which, at least in part, explained the pro-regenerative capacity of these cells in vivo [10]. Moreover, we and others observed that IL-10-treated primary human MΦ adopted an iron-releasing phenotype with consequences for tumor cell proliferation in vitro [44,45]. In the present study, we not only confirmed the anti-inflammatory and iron-releasing phenotype in primary murine MΦ but could also determine enhanced iron-loaded Lcn-2 in the MΦ-supernatant. Considering that both the release of iron as well as the production and secretion of Lcn-2 is a biological response of IL-10-treatment in MΦ, we hypothesized that MΦ-delivered iron-bound Lcn-2 accounts for mTEC recovery from Cisplatin-induced injury in vitro. The application of MΦ-conditioned media from both wt and Lcn-2^−/−^ mice to injured primary mTEC as well as rescue experiments applying iron-loaded recombinant Lcn-2 protein confirmed this speculation. Notably, we could detect a positive correlation between total intracellular iron amount in mTEC lysates and their proliferation, which, in turn, reinforced this assumed link between availability of iron-bound Lcn-2 and its release by anti-inflammatory MΦ. These findings suggest a direct implication of MΦ-Lcn-2 in main cytoprotective mechanisms during Cisplatin-induced renal injury. The above findings match with recent evidences implying that Lcn-2 functions as an additional alternative iron transporter within the tissue microenvironment [46,47].

In sum, we could show that mTEC are able to increase their intracellular iron pool by taking up MΦ-secreted iron-loaded Lcn-2, whereby proliferation and epithelial cell polarity is promoted. Still, not only mechanistic insights regarding Lcn-2 downstream signaling in renal epithelial cells is lacking, but also how iron is recycled and used in renal cells to promote recovery. Therefore, the understanding of molecular and genetic mechanisms that control Lcn-2 signaling and recycling of iron could offer new perspectives for future therapeutic avenues in acute kidney injury and progressive interstitial fibrosis.

## 4. Materials and Methods

### 4.1. Animals

C57BL/6 wt and Lcn-2^−/−^ mice with C57BL/6 background (both bred at mfd diagnostics, Wendelsheim, Germany) were kept in the central research facility of the university hospital Frankfurt. They were housed with water and food ad libitum in rooms with a 12h light cycle. Organ removal and animal care were performed in accordance with the “Guide for the care and use of laboratory animals“ (National Institutes of Health, volume 25, no. 28, revised 1996), EU Directive 86/609 EEC, and German Protection of Animals Act. No additional animal ethics approval is needed for organ removal for subsequent isolation of primary murine cells.

### 4.2. Isolation and Culture of Murine Proximal Tubular Epithelial Cells

Murine primary tubular epithelial cells (mTECs) were isolated from above described wt and Lcn-2^−/−^ mice as previously described [48,49]. In brief, after kidney removal, the tissue was minced and digested with a collagenase/dispase suspension. The digested fragments were passed through a 100 and 70 µm mesh. Subsequently cells caught with a 40 µm mesh were isolated and grown in DMEM/HAM’s F12 (1:1) with GlutaMAX (31331-028, Gibco, obtained from Thermo Fisher, Dreieich, Germany), supplemented with 10% FCS and 1% Penicillin/Streptomycin at 37 °C and 5% CO_2_ in a humidified atmosphere.

### 4.3. Establishment of Cisplatin Injury in mTECs Model

Cisplatin (cis-diamminedichloroplatinum II) is commonly used for chemotherapy in a wide variety of tumors [50]. However, it nonetheless has nephrotoxicity as a major side effect and limiting factor in clinical practice [51]. Mechanistically, Cisplatin causes primarily tubular necrosis and apoptosis [26,27,28] in a dose-and duration-dependent manner in both in vitro and in vivo [26,52]. Therefore, we established an in vitro cell culture model using cisplatin as a well-accepted clinically relevant model (Figure 1A). Viability of mTECs was determined by a photometric assay using 2,3-Bis-(2-Methoxy-4-Nitro-5-Sulfophenyl)-2H-Tetrazolium-5-Carboxanilide (XTT). In brief, subconfluent cells in 96-well plates were exposed to Cisplatin (Teva^®^, Ulm, Germany (stock solution 1 mg/mL (3.3 mM)) for 24 h or 48 h. Hereafter, XTT reagent was added to each well as described by the manufacturer (A8088, Applichem, Darmstadt, Germany) and incubated at 37 °C. Absorbance was measured in a microplate reader (ELx808, Bio-TEC Instruments Inc., Bad Friedrichshall, Germany) at 450 nm vs. 630 nm. Experiments were conducted in triplicate, sixfold, or ninefold in one to three independent experimental settings and are represented as mean ± SEM. The value of viability is expressed as percentage of viability of untreated control cells set as 100%.

### 4.4. Generation of Murine BMDM and Generation of Conditioned Media

Murine BMDM were generated by isolating the bone marrow of above described wt and Lcn-2^−/−^ mice. Cells were differentiated directly in 6-well plates (6 × 106 cells/well) in the presence of 20 ng/mL macrophage colony-stimulating factor (M-CSF) (Peprotech, Hamburg, Germany) for up to 7 days. At day 3, fresh M-CSF was added. For 24 h prior to stimulation cells were serum-starved. BMDM were stimulated for 24 h in RPMI medium supplemented with 20 ng/mL IL-10 (Peprotech, Hamburg, Germany) for the induction of an anti-inflammatory, iron-release phenotype as previously published [36]. The conditioned media of polarized BMDM were collected, centrifuged at 1000× *g* for 5 min, and aliquots were stored at −80 °C until further use. Supernatant of unstimulated MΦ served as control. Where indicated, iron chelator 2′2 DPD was added to wt cm at a concentration of 100 µM for 24 h.

### 4.5. Generation of Recombinant Lcn-2

Recombinant mouse Lcn-2 was produced by transformation of *E. coli* with a pGEX-4T-3-NGAL plasmid as already described for human recombinant Lcn-2 [21]. In order to test efficient Lcn-2-catechol-iron complex formation, UV-visible spectroscopy (UV-vis) was used as previously described [44]. To generate the holo-Lcn-2 protein equimolar amounts of 10 µM Lcn-2 were incubated with 10 µM catechol (Sigma-Aldrich, Steinheim, Germany) and 10 µM iron (Sigma).

### 4.6. Lcn-2 Immunoprecipitation

For immunoprecipitation (IP), supernatants of stimulated BMDMs were collected as described above. Dynabeads (Thermo Fisher, Dreieich, Germany) were added, and 1mg protein was incubated overnight at 4 °C in the presence of a specific antibody against Lcn-2 (MAB1857, R&D, Wiesbaden, Germany). Beads were precipitated using the DynaMag-2 magnet (Thermo Fisher, Dreieich, Germany) and washed three times with IP buffer. Protein was eluted by addition of 2× loading buffer and incubated at 95 °C for 5 min.

### 4.7. Atomic Absorption Spectrometry

Wt or Lcn-2^−/−^ MΦ were stimulated with IL-10 (20 ng/mL) for 24 h. Afterwards, mTEC were stimulated for 24 h with cm from either wt or Lcn-2^−/−^ MΦ. Where indicated, holo-Lcn-2 (1 µg/mL) was added to Lcn-2^−/−^ media for rescue experiments. The iron content of MΦ supernatants as well as the intracellular iron amount of stimulated mTECs was determined by graphite furnace atomic absorption spectrometry (AAS). Samples were measured as triplicates with a PinAAcleTM 900 T Atomic Absorption Spectrometer (PerkinElmer, Rodgau, Germany). Slit 0.2 nm and wavelength 248.33 nm were used as spectrometer parameters. A hollow cathode iron lamp (30 mA maximum operating current) was run at 100% maximum current. The calibration solutions (10 µg/L to 90 µg/L) were prepared by adequate dilution of iron standard for AAS (Sigma-Aldrich, Steinheim, Germany) stock solution. A pyrolysis temperature of 1400 °C and an atomization temperature of 2100 °C were used.

### 4.8. Establishment of Rescue Model Following Cisplatin Injury in mTECs

mTECs were either cultured in standard medium without FCS (designated as ctrl) or injured with 5 µM Cisplatin (designated as Cis) for 24 h. Hereafter, Cisplatin-injured mTECs were cultured for a further 24 h in cm from IL-10-stimulated MΦ from wt (designated as cm wt MΦ) or Lcn-2^−/−^ mice (designated as cm Lcn-2^−/−^ MΦ). For rescue experiments, cm from Lcn-2^−/−^ was supplemented with holo-Lcn-2 (designated as holo-Lcn-2).

### 4.9. RNA Extraction and Quantitative Real-Time PCR (qPCR)

RNA isolation cDNA synthesis and qPCR were performed as previously described [46]. Briefly, RNA was isolated (30-1010, peqlab, Erlangen, Germany) and transcribed into cDNA (K1642, Thermo Fisher), serving as template in qPCR mix (1725006CUST, Bio-Rad, Dreieich, Germany). We used TBP (TATA-binding protein) as an internal housekeeping gene control for detection of gene expression in BMDM, while RPS27a (ribosomal protein 27a) mRNA expression served as a housekeeping gene for real-time (RT)-PCR and β-actin for qPCR analysis in mTEC [53]. Primers were bought from Bio-Rad, Biomers (Ulm, Germany), or Thermo Fisher and are listed in Table 1.

### 4.10. Western Blot

Protein samples of 5 μg were dissolved in sample buffer (Laemmli buffer, Bio-Rad) containing DTT (dithiothreitol) and treated for 5 min at 95 °C. Protein samples were separated on a 4–12% CRIT XT BIS-TRIS GEL (Bio-Rad) and transferred to a PVDF membrane (Bio-Rad). Membranes were blocked with 5% fat-free milk in tris-buffered saline containing 0.1% Tween-20 for 1.5 h. Primary antibodies were added and membranes were incubated overnight at 4 °C. Hereafter, an adequate horseradish peroxidase (HRP)-conjugated secondary antibodies were added and incubated for 2 h at room temperature. Visualization was performed using Clarity ECL or Clarity ECL max (Biorad). Primary Antibodies: Klotho (MAB1819, 1:500, R&D), KIM-1 (#3809, 1:1000, ProSci, Poway, CA, USA), Pan-Actin antibody (#4968, dilution, Cell Signaling, Frankfurt am Main, Deutschland).

### 4.11. Lcn-2 ELISA

Supernatants were collected from cultured MΦ. A volume of 100 µL of each sample was applied to an ELISA well-plate previously covered with the anti-Lcn-2 (MAB1857, R&D) and blocked for 1 h. After sample incubation, the detection anti-Lcn-2 antibody was added. HRP-conjugated avidin (Invitrogen, obtained from Thermo Fisher) was incubated for 1h, the color reagent (OPD tablets; Dako, obtained from Agilent, Waldbronn, Germany) was added, and the color development was assessed.

### 4.12. Phalloidin-Staining

Changes in the cytoskeleton and the F-actin stress fibers were visualized by phalloidin staining. Briefly, cells were fixed in 4% buffered formaldehyde for 10 min and then permeabilized with PBS containing 0.1% Triton X-100 and 1% BSA for 30 min. The slides were then incubated with Alexa Fluor 568 phalloidin (00027, dilution 1:40, Molecular Probes Inc.) in PBS with 1% BSA for 30 min, counterstained with DAPI (D9542, Sigma-Aldrich, Steinheim, Germany), washed three times with PBS, and finally mounted using mowiol (Calbiochem, Darmstadt, Germany). Images were acquired on an LSM 800 (Zeiss, Wetzlar, Germany) confocal microscope.

### 4.13. Immunofluoresecence Cytokeratin Stain

Confluent monolayers were rinsed three times with PBS and fixed with ice-cold methanol/acetone (1:1) for 5 min. The fixed cells were washed twice. Unspecific binding sites were blocked by PBS containing 5% normal goat serum for 20 min. Primary antibody (anti-Pan Cytokeratin C2931, 1:400, Sigma-Aldrich, Steinheim, Germany) was applied and incubated for 30 min at 37 °C with gentle shaking. After washing, cells were incubated with a Cy3-conjugated goat-anti-mouse IgG mAb for 30 min at 37 °C. Slides were mounted with mounting medium and examined using Zeiss fluorescence microscope equipment.

### 4.14. xCELLigence Proliferation Assay

Proliferation of mTECs was measured using the RTCA DP xCELLigence instrument (OLS, Bremen, Germany) as described previously [20]. Data are presented as the slope per hour (slope 1/h) of the normalized cell index as a measure for the time-dependent changes in impedance.

### 4.15. Statistical Analyses

Statistical analyses were performed applying GraphPad Prism^®^ 5.02 software (GraphPad Software, San Diego, CA, USA). The distribution of variables was tested for normality using the Kolmogorov–Smirnov test. Accordingly, statistical significance was calculated using one-way ANOVA followed by Tukey’s multiple comparison test or Kruskal–Wallis test followed by Dunn’s posthoc test, where applicable. Significance of correlations was determined by Spearman’s test including all investigated groups. *p*-values ≤ 0.05 were assumed as statistically significant. In the figures, horizontal lines within the boxes represent the medians, boxes represent the interquartile range (25–75%). Whiskers above and below the box indicate the 90th and 10th percentiles. The individual points that are plotted beyond the whiskers represent outliers, which were included in the statistical analyses.

## Figures and Tables

**Table 1:** Primer sequences.

Primer	Sequence
β-actin	Forward: 5-CCACCATGTACCCAGGCATT-3 Reverse: 5-AGGGTGTAAAACGCAGCTCA-3
β-Catenin	Forward: 5-TCTGAGGACAAGCCACAGGATTACA-3 Reverse: 5-GGGCACCAATGTCCAGTCCAA-3
CK18	Forward: 5-TTGTCACCACCAAGTCTGCC-3 Reverse: 5-TTGTATCGGGCCTCCACATC-3
E-Cadherin (real-time)	Forward: 5-TGAAGAAGGAAGAAGA-3 Reverse: 5-TGGGAGCCACTTTCGA-3
E-Cadherin (qRT)	Forward: CAACGATCCTGACCAGCAGT Reverse: TGTATTGCTGCTTGGCCTCA
IL-1β	Forward: 5-AGGCCACAGGTATTTTGTCG-3 Reverse: 5-GACCTTCCAGGATGAGGACA-3
iNOS	Forward: 5-ACCCTAAGAGTCACAAAATGGC-3 Reverse: 5-TTGATCCTCACATACTGTGGACG-3
Lcn-2	qHsaCED0045408
MRC	Forward: 5-GGAGTGATGGAACCCCAGTG-3 Reverse: 5-CTGTCCGCCCAGTATCCATC-3
PCNA	Forward: 5-AATGGGGTGAAGTTTTCTGC-3 Reverse: 5-CAGTGGAGTGGCTTTTGTGA-3
RPS27a	Forward: 5-GACCCTTACGGGGAAAACCAT-3 Reverse: 5-AGACAAAGTCCGGCCATCTTC-3
Stathmin	Forward: 5-CTTGCGAGAGAAGGACAAGC-3 Reverse: 5-CGGTCCTACATCGGCTTCTA-3
TBP	Forward: 5-GGGCCGCCGGTTAACT-3 Reverse: 5-AGCCCTGAGCGTGGCA-3
TNFα	Forward: 5-CCATTCCTGAGTTCTGCAAAGG-3 Reverse: 5-AGGTAGGAAGGCCTGAGATCTTATC-3
YM-1	Forward: 5-GGGCATACCTTTATCCTGAG-3 Reverse: 5-CCACTGAAGTCATCCATGTC-3
ZO-1 (real-time)	Forward: 5-GCCATTACACGGTCCTCTGA-3 Reverse: 5-GCGAAAGGTAAGGGACTGG-3
ZO-1 (qPCR)	Forward: GCCATTACACGGTCCTCTGA Reverse: GCGAAAGGTAAGGGACTGGA

## Supplementary Materials

Supplementary materials can be found at https://www.mdpi.com/1422-0067/21/6/2038/s1.

## Abbreviations

AAS	Atomic absorption spectrometry
BMDM	Bone marrow-derived macrophages
Cis	Cisplatin
Cm	Conditioned medium
ctrl	Control
IL-10	Interleukin 10
IRI	Ischemia-reperfusion injury
KIM-1	Kidney injury molecule 1
Lcn-2	Lipocalin-2
mTEC	Murine tubular epithelial cells
MΦ	Macrophages
wt	Wildtype

## References

[1] Urbschat A., Obermuller N., Haferkamp A. (2011). Biomarkers of kidney injury. Biomarkers.

[2] Ronco C., Bellomo R., Kellum J.A. (2019). Acute kidney injury. Lancet.

[3] Bonventre J.V. (2003). Dedifferentiation and proliferation of surviving epithelial cells in acute renal failure. J. Am. Soc. Nephrol..

[4] Huen S.C., Cantley L.G. (2017). Macrophages in Renal Injury and Repair. Annu. Rev. Physiol..

[5] Ysebaert D.K., De Greef K.E., Vercauteren S.R., Ghielli M., Verpooten G.A., Eyskens E.J., De Broe M.E. (2000). Identification and kinetics of leukocytes after severe ischaemia/reperfusion renal injury. Nephrol. Dial. Transpl..

[6] Vinuesa E., Hotter G., Jung M., Herrero-Fresneda I., Torras J., Sola A. (2008). Macrophage involvement in the kidney repair phase after ischaemia/reperfusion injury. J. Pathol..

[7] Mosser D.M., Edwards J.P. (2008). Exploring the full spectrum of macrophage activation. Nat. Rev. Immunol..

[8] Labonte A.C., Tosello-Trampont A.C., Hahn Y.S. (2014). The role of macrophage polarization in infectious and inflammatory diseases. Mol. Cells.

[9] Jung M., Brune B., Hotter G., Sola A. (2016). Macrophage-derived Lipocalin-2 contributes to ischemic resistance mechanisms by protecting from renal injury. Sci. Rep..

[10] Jung M., Sola A., Hughes J., Kluth D.C., Vinuesa E., Vinas J.L., Perez-Ladaga A., Hotter G. (2012). Infusion of IL-10-expressing cells protects against renal ischemia through induction of lipocalin-2. Kidney Int..

[11] Sola A., Weigert A., Jung M., Vinuesa E., Brecht K., Weis N., Brune B., Borregaard N., Hotter G. (2011). Sphingosine-1-phosphate signalling induces the production of Lcn-2 by macrophages to promote kidney regeneration. J. Pathol..

[12] Recalcati S., Locati M., Cairo G. (2012). Systemic and cellular consequences of macrophage control of iron metabolism. Semin. Immunol..

[13] Haase M., Devarajan P., Haase-Fielitz A., Bellomo R., Cruz D.N., Wagener G., Krawczeski C.D., Koyner J.L., Murray P., Zappitelli M. (2011). The outcome of neutrophil gelatinase-associated lipocalin-positive subclinical acute kidney injury: A multicenter pooled analysis of prospective studies. J. Am. Coll. Cardiol..

[14] Paragas N., Qiu A., Zhang Q., Samstein B., Deng S.X., Schmidt-Ott K.M., Viltard M., Yu W., Forster C.S., Gong G. (2011). The Ngal reporter mouse detects the response of the kidney to injury in real time. Nat. Med..

[15] Mishra J., Ma Q., Prada A., Mitsnefes M., Zahedi K., Yang J., Barasch J., Devarajan P. (2003). Identification of neutrophil gelatinase-associated lipocalin as a novel early urinary biomarker for ischemic renal injury. J. Am. Soc. Nephrol..

[16] Mishra J., Mori K., Ma Q., Kelly C., Barasch J., Devarajan P. (2004). Neutrophil gelatinase-associated lipocalin: A novel early urinary biomarker for cisplatin nephrotoxicity. Am. J. Nephrol..

[17] Mishra J., Mori K., Ma Q., Kelly C., Yang J., Mitsnefes M., Barasch J., Devarajan P. (2004). Amelioration of ischemic acute renal injury by neutrophil gelatinase-associated lipocalin. J. Am. Soc. Nephrol..

[18] Mori K., Lee H.T., Rapoport D., Drexler I.R., Foster K., Yang J., Schmidt-Ott K.M., Chen X., Li J.Y., Weiss S. (2005). Endocytic delivery of lipocalin-siderophore-iron complex rescues the kidney from ischemia-reperfusion injury. J. Clin. Investig..

[19] Gwira J.A., Wei F., Ishibe S., Ueland J.M., Barasch J., Cantley L.G. (2005). Expression of neutrophil gelatinase-associated lipocalin regulates epithelial morphogenesis in vitro. J. Biol. Chem..

[20] Jung M., Oren B., Mora J., Mertens C., Dziumbla S., Popp R., Weigert A., Grossmann N., Fleming I., Brune B. (2016). Lipocalin 2 from macrophages stimulated by tumor cell-derived sphingosine 1-phosphate promotes lymphangiogenesis and tumor metastasis. Sci. Signal..

[21] Oren B., Urosevic J., Mertens C., Mora J., Guiu M., Gomis R.R., Weigert A., Schmid T., Grein S., Brune B. (2016). Tumour stroma-derived lipocalin-2 promotes breast cancer metastasis. J. Pathol..

[22] Vinuesa E., Sola A., Jung M., Alfaro V., Hotter G. (2008). Lipocalin-2-induced renal regeneration depends on cytokines. Am. J. Physiol. Ren. Physiol..

[23] Devireddy L.R., Gazin C., Zhu X., Green M.R. (2005). A cell-surface receptor for lipocalin 24p3 selectively mediates apoptosis and iron uptake. Cell.

[24] Paller M.S., Hedlund B.E. (1988). Role of iron in postischemic renal injury in the rat. Kidney Int..

[25] Paller M.S., Hedlund B.E. (1994). Extracellular iron chelators protect kidney cells from hypoxia/reoxygenation. Free Radic. Biol. Med..

[26] Lieberthal W., Triaca V., Levine J. (1996). Mechanisms of death induced by cisplatin in proximal tubular epithelial cells: Apoptosis vs. necrosis. Am. J. Physiol..

[27] Borch R.F., Pleasants M.E. (1979). Inhibition of cis-platinum nephrotoxicity by diethyldithiocarbamate rescue in a rat model. Proc. Natl. Acad. Sci. USA.

[28] Zhou H., Kato A., Yasuda H., Miyaji T., Fujigaki Y., Yamamoto T., Yonemura K., Hishida A. (2004). The induction of cell cycle regulatory and DNA repair proteins in cisplatin-induced acute renal failure. Toxicol. Appl. Pharm..

[29] Kashiwagi E., Tonomura Y., Kondo C., Masuno K., Fujisawa K., Tsuchiya N., Matsushima S., Torii M., Takasu N., Izawa T. (2014). Involvement of neutrophil gelatinase-associated lipocalin and osteopontin in renal tubular regeneration and interstitial fibrosis after cisplatin-induced renal failure. Exp. Toxicol. Pathol..

[30] Molitoris B.A., Marrs J. (1999). The role of cell adhesion molecules in ischemic acute renal failure. Am. J. Med..

[31] Bonventre J.V., Kelly K.J. (1996). Adhesion molecules and acute renal failure. Adv. Nephrol. Necker Hosp..

[32] Kruidering M., van de Water B., Zhan Y., Baelde J.J., Heer E., Mulder G.J., Stevens J.L., Nagelkerke J.F. (1998). Cisplatin effects on F-actin and matrix proteins precede renal tubular cell detachment and apoptosis in vitro. Cell Death Differ..

[33] Jung M., Hotter G., Vinas J.L., Sola A. (2009). Cisplatin upregulates mitochondrial nitric oxide synthase and peroxynitrite formation to promote renal injury. Toxicol. Appl. Pharm..

[34] Curmi P.A., Gavet O., Charbaut E., Ozon S., Lachkar-Colmerauer S., Manceau V., Siavoshian S., Maucuer A., Sobel A. (1999). Stathmin and its phosphoprotein family: General properties, biochemical and functional interaction with tubulin. Cell Struct. Funct..

[35] Peschanski M., Hirsch E., Dusart I., Doye V., Marty S., Manceau V., Sobel A. (1993). Stathmin: Cellular localization of a major phosphoprotein in the adult rat and human CNS. J. Comp. Neurol..

[36] Mertens C., Akam E.A., Rehwald C., Brune B., Tomat E., Jung M. (2016). Intracellular Iron Chelation Modulates the Macrophage Iron Phenotype with Consequences on Tumor Progression. PLoS ONE.

[37] Recalcati S., Locati M., Marini A., Santambrogio P., Zaninotto F., De Pizzol M., Zammataro L., Girelli D., Cairo G. (2010). Differential regulation of iron homeostasis during human macrophage polarized activation. Eur. J. Immunol..

[38] Rehwald C., Schnetz M., Urbschat A., Mertens C., Meier J.K., Bauer R., Baer P., Winslow S., Roos F.C., Zwicker K. (2019). The iron load of lipocalin-2 (LCN-2) defines its pro-tumour function in clear-cell renal cell carcinoma. Br. J. Cancer.

[39] Mertens C., Mora J., Oren B., Grein S., Winslow S., Scholich K., Weigert A., Malmstrom P., Forsare C., Ferno M. (2018). Macrophage-derived lipocalin-2 transports iron in the tumor microenvironment. Oncoimmunology.

[40] Baer P.C., Nockher W.A., Haase W., Scherberich J.E. (1997). Isolation of proximal and distal tubule cells from human kidney by immunomagnetic separation. Technical note. Kidney Int..

[41] Dekel B., Zangi L., Shezen E., Reich-Zeliger S., Eventov-Friedman S., Katchman H., Jacob-Hirsch J., Amariglio N., Rechavi G., Margalit R. (2006). Isolation and characterization of nontubular sca-1+lin- multipotent stem/progenitor cells from adult mouse kidney. J. Am. Soc. Nephrol..

[42] Dasari S., Tchounwou P.B. (2014). Cisplatin in cancer therapy: Molecular mechanisms of action. Eur. J. Pharm..

[43] Volarevic V., Djokovic B., Jankovic M.G., Harrell C.R., Fellabaum C., Djonov V., Arsenijevic N. (2019). Molecular mechanisms of cisplatin-induced nephrotoxicity: A balance on the knife edge between renoprotection and tumor toxicity. J. Biomed. Sci..

[44] Zahedi K., Wang Z., Barone S., Tehrani K., Yokota N., Petrovic S., Rabb H., Soleimani M. (2004). Identification of stathmin as a novel marker of cell proliferation in the recovery phase of acute ischemic renal failure. Am. J. Physiol. Cell Physiol..

[45] Miura K., Goldstein R.S., Pasino D.A., Hook J.B. (1987). Cisplatin nephrotoxicity: Role of filtration and tubular transport of cisplatin in isolated perfused kidneys. Toxicology.

[46] Witzgall R., Brown D., Schwarz C., Bonventre J.V. (1994). Localization of proliferating cell nuclear antigen, vimentin, c-Fos, and clusterin in the postischemic kidney. Evidence for a heterogenous genetic response among nephron segments, and a large pool of mitotically active and dedifferentiated cells. J. Clin. Investig..

[47] Caracausi M., Piovesan A., Antonaros F., Strippoli P., Vitale L., Pelleri M.C. (2017). Systematic identification of human housekeeping genes possibly useful as references in gene expression studies. Mol. Med. Rep..

[48] Jung M., Weigert A., Tausendschon M., Mora J., Oren B., Sola A., Hotter G., Muta T., Brune B. (2012). Interleukin-10-induced neutrophil gelatinase-associated lipocalin production in macrophages with consequences for tumor growth. Mol. Cell Biol..

[49] Tanase D.M., Gosav E.M., Radu S., Costea C.F., Ciocoiu M., Carauleanu A., Lacatusu C.M., Maranduca M.A., Floria M., Rezus C. (2019). The Predictive Role of the Biomarker Kidney Molecule-1 (KIM-1) in Acute Kidney Injury (AKI) Cisplatin-Induced Nephrotoxicity. Int. J. Mol. Sci..

[50] Mitobe M., Yoshida T., Sugiura H., Shirota S., Tsuchiya K., Nihei H. (2005). Oxidative stress decreases klotho expression in a mouse kidney cell line. Nephron Exp. Nephrol..

[51] Sugiura H., Yoshida T., Tsuchiya K., Mitobe M., Nishimura S., Shirota S., Akiba T., Nihei H. (2005). Klotho reduces apoptosis in experimental ischaemic acute renal failure. Nephrol. Dial. Transpl..

[52] Hu M.C., Shi M., Zhang J., Quinones H., Kuro-o M., Moe O.W. (2010). Klotho deficiency is an early biomarker of renal ischemia-reperfusion injury and its replacement is protective. Kidney Int..

[53] Duffield J.S. (2011). Macrophages in kidney repair and regeneration. J. Am. Soc. Nephrol..

