# Supplementary material for: Macrophage-Secreted Lipocalin-2 Promotes Regeneration of Injured Primary Murine Renal Tubular Epithelial Cells
Source: Int J Mol Sci. 2020 Mar 16;21(6):2038. doi: 10.3390/ijms21062038 (PMC7139578; doi:10.3390/ijms21062038)
Supplement: Supplementary file 1 [file ijms-21-02038-s001.pdf]

International Journal of
Molecular Sciences

Article

Supplementary Materials: Macrophage-secreted lipocalin-2 promotes regeneration of injured primary murine renal tubular epithelial cells

Anja Urbschat [1], Anne-Kathrin Thiemens [2], Christina Mertens [3], Claudia Rehwald [3], Julia K. Meier [3], Patrick C. Baer [2,#] and Michaela Jung [3,*,#]

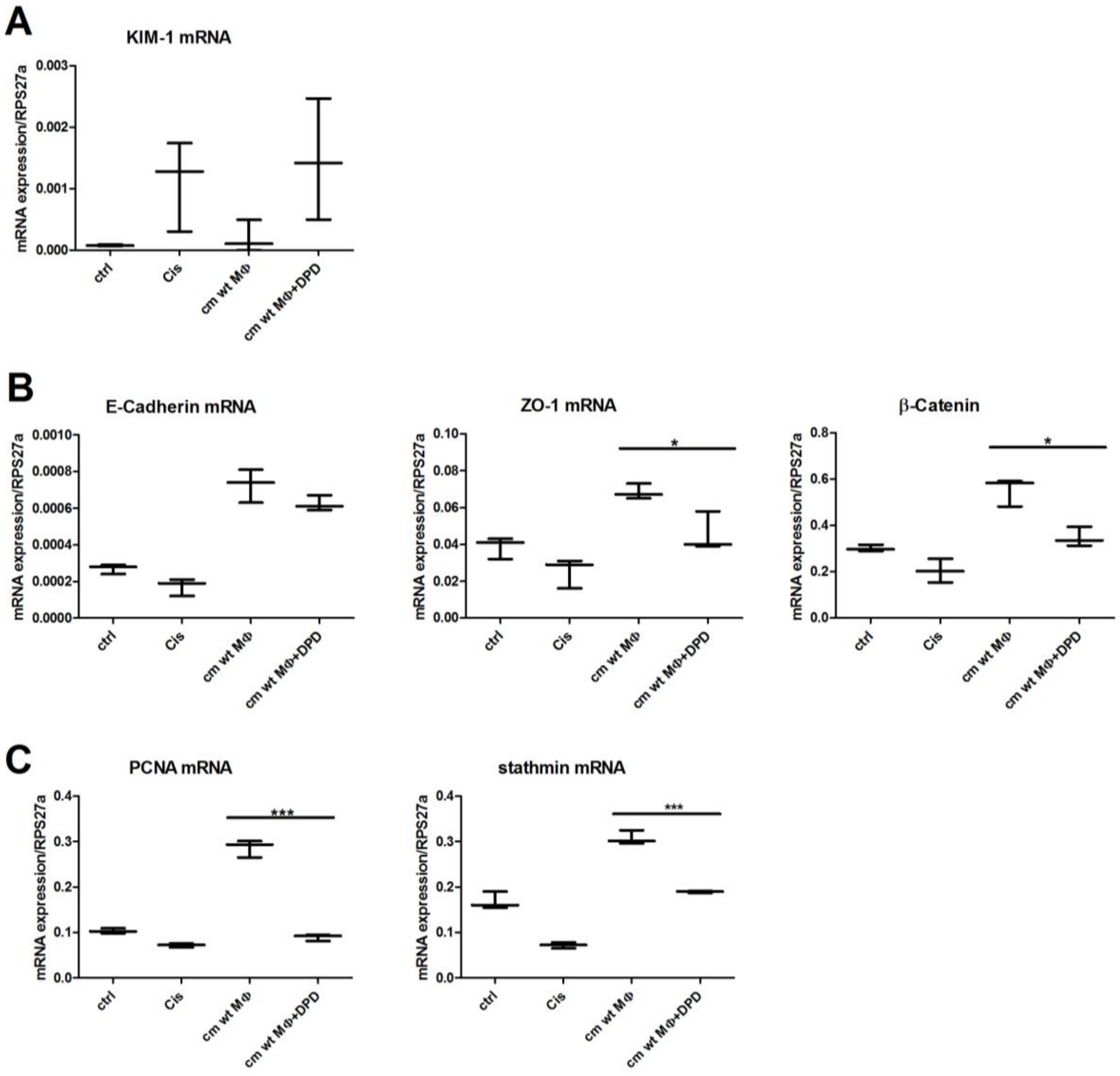

Supplemental Figure 1: Addition of iron chelator 2'2 DPD blocks the protective effects of macrophage supernatants

*mRNA expression relative to the housekeeping gene RPS27a of the (A) injury marker KIM-1, (B) the epithelial phenotype marker E-Cadherin, β-catenin, and Zonula occludens-1 (ZO-1) as well as (C) proliferation markers PCNA and stathmin. .*p<0.05, ***p<0.001 (n=3; t-test).*
